# How could the United Nations Global Digital Compact prevent cultural imposition and hermeneutical injustice?

**DOI:** 10.1016/j.patter.2024.101078

**Published:** 2024-11-08

**Authors:** Arthur Gwagwa, Warmhold Jan Thomas Mollema

**Affiliations:** 1 Ethics Institute, Department of Philosophy & Religious Studies, Utrecht University, Janskerkhof 13, 3512 BL Utrecht, the Netherlands; 2 Department of Information and Computing Sciences, Utrecht University, Princetonplein 5, 3584 CC Utrecht, the Netherlands

**Keywords:** AI governance, AI regulation, Global Digital Compact, United Nations, cultural imposition, hermeneutical injustice, domination, artificial intelligence, AI ethics, AI colonialism

## Abstract

As the geopolitical superpowers race to regulate the digital realm, their divergent rights-centered, market-driven, and social-control-based approaches require a global compact on digital regulation. If diverse regulatory jurisdictions remain, forms of domination entailed by cultural imposition and hermeneutical injustice related to AI legislation and AI systems will follow. We argue for consensual regulation on shared substantive issues, accompanied by proper standardization and coordination. Failure to attain consensus will fragment global digital regulation, enable regulatory capture by authoritarian powers or bad corporate actors, and deepen the historical geopolitical power asymmetries between the global South and the global North. To prevent an unjust regulatory landscape where the global South’s cultural and hermeneutic resources are absent, two principles for the Global Digital Compact to counter these prospective harms are proposed and discussed: (1) “recognitive consensus on key substantive benefits and harms” and (2) “procedural consensus on global coordination and essential standards.”

## The bigger picture

This paper is a contribution to the discourse surrounding the United Nations’ Global Digital Compact (GDC) to be agreed at the Summit of the Future in September 2024 that has come forth out of the Secretary-General’s report “Our Common Agenda.” Its critical note is that the GDC should not merely reflect the digital regulatory superpowers’ interests, goals, and problems with respect to AI and digital technologies but also incorporate and be accommodated to those of the global South—geographical regions such as Sub-Saharan Africa and Latin America that are currently subverted on the global stage for digital regulation. Based firstly on a conception of what digital regulation is, secondly on accounts of the United States’, European Union’s, and China’s diverse regulatory directions, and thirdly on the forms of domination that arise when the regulation of the digital realm is imposed, this paper proposes two critical and aspirational principles for the GDC. The first is “recognitive consensus on key substantive benefits and harms,” and the second is “procedural consensus on global coordination and essential standards.” Our formulation of both emphasizes firstly how the current discussion is skewed to a few dominant approaches to global AI governance and digital regulation. We show how the formulation of the principles in the global compact can prevent, on the one hand, the cultural imposition of superpowers’ frameworks and values in the AI domain on the global South and, on the other hand, the disqualification of the experience of citizens of the global South from the global understanding of the risks and benefits of AI deployment. As global regulation of AI and the digital realm is increasingly being defined, we think it is of the essence to shed light on its lack of inclusion of perspectives from the global periphery.

## Teaser

In this paper, authors discuss the UN Global Digital Compact and argue for a global consensus at procedural and substantive levels on how diverse regions could standardize their approaches to AI harms, risks, and benefits. This urgent need would align their varied philosophical approaches to AI governance with the Compact’s objectives for an inclusive digital future for all humanity, anchored in universal human rights.

## Introduction

Following the political declaration adopted at the occasion of the United Nations’ (UN) 75th anniversary in September 2020, the Secretary-General in September 2021 released his report “Our Common Agenda.” The Common Agenda proposes a Global Digital Compact (GDC) to be agreed at the Summit of the Future in September 2024 through a technology track involving all stakeholders: governments, the UN system, the private sector (including tech companies), civil society, grass-roots organizations, academia, and individuals, including youth. This paper argues that although a broad compact on digital regulation may not be plausible or attainable, there is an increasing global need for a broad consensus on shared issues related to regulating AI technologies and their digital corollaries. This argument depends on a broad definition of digital regulations as a societal effort to channel digital technologies in the public interest and encompass all forms of governance. The details and even the principles of any such global consensus (particularly in the current UN institutional embedding) remain to be spelled out. Still, the current indications must make it plausible that consensual discussions toward globally justified rules are possible. We argue that the GDC is a good starting point (i.e., might be appropriate) and institutional carrier for global AI regulations (https://www.un.org/en/content/common-agenda-report/). Firstly, it is aimed at avoiding Internet fragmentation and, although it may not confer legal rights, its message is realistic and not just aspirational and symbolic. In terms of principles, it offers a normative counterpoint to the fragmentation inherited from the first decades of the Internet age that is now reflected in the divergent digital governance proposals. Secondly, it articulates the global desire and drive to establish common ethical standards of behavior applicable to all humankind in the digital realm, as expressed at the Bletchley and Davos Summits. Thirdly and finally, as it is UN-led, it also offers the possibility of reinforcing the current international human rights standards that all the UN member states agreed on at the World Conference on Human Rights in Vienna in 1993. However, we are not suggesting that this consensus is to replace the current multi-stakeholder approach to Internet governance based on an “open” Internet and a tightly restricted role for governments versus a centralized, state-led form of governance under the auspices of the UN (and thus state-controlled). At the same time, a multi-stakeholder consensus will ensure adherence to international law and not the rules-based global order often propagated by the US and its allies.

It is essential to regard the regulation of the Internet and AI as co-extensive because the latter increasingly features in and draws on the former. This paper aims to sketch the landscape of the current digital governance paths that different regions and “Western, educated, industrialized, rich, and democratic” (WEIRD) and non-WEIRD powerful AI economies are carving; it then offers a normative analysis of the current paths and reiterates the principles that underpin the GDC, i.e., the need to agree on substantive issues relating to shared harms and risks as well as coordination and standardization on the approach to such critical matters on common areas of concern, such as cross-border data sharing and beneficiation and a balanced approach to mitigating AI risks. We do not aim to be presumptuous in spelling out the principles, as these issues have been articulated before. Still, we offer conceptual and normative reflections on why the current unilateral approaches to digital and AI regulation may result in injustice, particularly cultural imposition and hermeneutical injustice in the global South. As the understanding of AI and its full capabilities, benefits, and risks is still evolving, the paper recommends that there should be international recognitive and procedural consensus on governance and on significant related issues that need coordination, as cross-border data flows anchored in international human rights norms and standards, to counter the injustices of cultural imposition and hermeneutical injustice in the global South.

Although our paper is mainly normative and conceptual, we highlight the dangers of the current geopolitical power asymmetries and formulate principles based on actual issues. Therefore, this paper will not formulate measures on the level of detail of concrete policies. However, the position defended here remains a critical starting point for developing such policies.

The structure of this paper is as follows. First, the challenges digital technologies and governance pose to society are sketched in broad strokes, particularly the disruptive capacity to realize the injustices of cultural imposition and hermeneutical injustice for the already marginalized. Second, we will develop and define what the concept of regulation means for the digital realm. Subsequently, the three main approaches to regulating the digital realm are reviewed: the European Union (EU), the US, and China. This is followed by a discussion of global legislation principles and the assertion that without an institutional embedding for globally discoursed consensual justification on AI rules, the realm of experience of social groups in the global South is excluded, and the groups are hermeneutically harmed.^1^ This discussion will set the stage for presenting two legislation principles for the GDC focused on recognizing and protecting social groups in the global South from cultural imposition and hermeneutical injustice related to AI. The paper concludes by discussing the merits of the principles and related challenges for the approach envisioned here.

## Challenges for the global governance of the digital sphere

A consensus regarding global digital governance on shared issues (common goods) is essential to avoid fragmentation and the possibility of regulatory capture by a few powerful states and bad corporate actors. Even if consensus is reached, diverse jurisdictions will remain. Additionally, existing human rights frameworks and norms (as fleshed out in special rapporteurs and treaty bodies, among others) should address issues and harms arising from digitalization, such as privacy, discrimination, bias, and other related rights. The lack of national, regional, and international consensus will primarily benefit the AI-producing countries while disadvantaging marginalized populations in countries that are sales markets for AI technologies, mainly in the global South. Those most significantly impacted by the lack of regulation are historically disadvantaged and marginalized groups and those who sit at their intersections, resulting in the amplification of these digital inequalities across the world.^2^ These groups are prone to technological domination, as their social relation with the global North is characterized by unequal access to power resources. Their economic vulnerabilities make them a group more prone to exploitation. Writing on technological disruption, Nickel et al. argue that correctly understanding the role of power differences requires attending to its epistemic dimension, as people in different positions may experience moral uncertainty quite differently (differential disruption).^3^^,^^4^ Following Nickel et al., we explicitly pursue the cultural and epistemic injustices related to the imposition of forms of AI legislation.

Even though a diversity of jurisdictions is necessary to avoid regulatory capture by a single legal entity, a lack of global consensus on key issues may impact the human rights of all people globally. It is a reasonable foresight that regulatory capture and a lack of consensus will disproportionately affect already vulnerable groups in digital spaces because the regulation is not attuned to their contexts and is ill-prepared for the possible ways AI technologies may harm them. Additionally, there is the effect of the invigoration of the geopolitical rivalry between the US and China (https://www.usip.org/publications/2022/12/10-things-know-about-us-china-rivalry-africa). Such rivalries have historically disadvantaged Africa, with global superpowers regarding Africa as “a theater of operations rather than the focus itself.”^5^ An international framework would offer an institutional embedding in which these disadvantaged groups and smaller countries pool their sovereign interests to negotiate with global powers, states, and corporations.

However, would the UN GDC adequately reflect an institutional embedding for consensual discussions on AI? The GDC, set up under the Office of the Secretary-General’s Envoy on Technology, “outlines shared principles for an open, free and secure digital future for all, including digital connectivity, avoiding Internet fragmentation, providing people with options as to how their data is used, application of human rights online, and promoting a trustworthy Internet by introducing accountability criteria for discrimination and misleading content” (https://www.un.org/techenvoy/). Before turning to whether the GDC is the suitable medium, it is essential to consider what forms of injustice are at stake for the global South, apart from the already much-talked-about economic dominance, biases, and information disorders caused by AI.

## The dangers of cultural imposition and hermeneutical injustice

Big Tech is not the saviour Africa needs to look up to, and its presence in Africa is driven primarily by profits, monopoly, and a rush to grab power more than anything else.^6^

Concerning AI, many scenarios deserve the label “worst-case.” However, be it the threat of the mindless “paper-clip” destruction of superintelligence, rogue artificial general intelligence (AGI), or information and digital hegemony enabled by a local concentration of advanced AI technologies, what is regarded as the “worst case” is determined by how the AI systems in question are compared to the biological prototype of intelligence. This evaluation is bound to be epistemically and culturally relative. For example, considering the thick concept of “intelligence,” the contents of an answer informed by Ubuntu-thought will differ significantly from how the question is answered in American AI development communities. Also, how some corporations envision the perfect application of AGI as digital companions fine-tuned to one’s life goals is an example of Western universalism regarding how life could be enhanced, which implicitly prescribes how life should be lived. We will return to some of these issues in the discussion. The point is that regardless of how one envisions future scenarios, the existing global power asymmetries yield that the harms of these dystopias inevitably involve two types of epistemic injustice, namely cultural imposition and hermeneutical injustice, diffracting how the dystopias’ central harms play out in different geographical regions.

Before defining these, however, consider a more realistic, less dystopian worst-case scenario. Suppose no international embedding for AI governance can achieve consensual justification. We are then left with an AI governance skewed toward the interests of WEIRD countries and corporations. The global South’s countries and marginalized social groups in the global North will be at the AI superpowers’ whim about how their rights and needs are conceptualized. Without a globally inclusive institutional embedding, these groups, the “wretched of AI,” to paraphrase Frantz Fanon,^7^ will form a legislative periphery, depending on a digital metropole. This metaphorical form of “AI colonialism” connects the two epistemic injustices.^8^ These dangers undergird the importance of attaining consensus. Still, they will also figure in different empirical manifestations of a “worst-case scenario” given the inevitable importing of the current South/North asymmetry in AI capabilities.

Firstly, we think of (1) the cultural imposition of having AI frameworks and regulations from the metropole forced on the periphery and (2) the hermeneutical injustice of peripherical perspectives being disqualified as legitimate perspectives on knowledge or ways of developing AI by the Western/capitalistic focus on the scientistic epistemologies that are embedded in and propagated by AI, particularly machine learning models. We argue these injustices and their interrelations are worth considering because they are not “stand-alone” but amount to shared forms of domination, undermining the agency of people belonging to the aforementioned social groups. Following Philip Pettit’s republican conception of domination, we can say that one does not have to be conscious of one’s choice set being constrained to be dominated. All that is necessary is that choice sets are restricted and that the agency enabling that is arbitrarily and non-reciprocally channeled. Likewise, one should distinguish violations (i.e., intentional restrictions) from unintentional vitiations (i.e., accidentally making unavailable) of these choice sets.^9^ Schematically, the duo of cultural imposition and hermeneutical injustice results in the following abstract forms of domination.

(1) When using AI systems, prefigured configurations of AI models or user interactions are imposed top-down from a cultural and hermeneutical standpoint, restricting how users interact with these AI systems. They constrain and shape the exercise of users’ capacities as citizens because AI systems situate or mediate contributions to the public sphere. Because AI providers centralize control over and prefigure future applications of their models, this violates the receiving users’ choice sets. This results in what Uğur Aytaç has called digital domination in the context of social media platforms.^10^
(2) If unregulated, Western AI systems will increasingly become unavoidable for exercising and developing public capabilities, just as has been the case with Internet provision and social media. This involves many vitiations of citizens’ choice sets because developing their models is too costly in a globally dominated market.^11^ Therefore, they are bound to swallow these AI systems’ cultural and epistemological configurations. Thereby, the expression of indigenous sense making is suppressed, and hermeneutically differing approaches to justifying knowledge are disqualified.
(3) Lastly and most generally, cultural imposition and hermeneutical injustice vitiate choice sets by forcing cognitive or conceptual resources to become “unavailable” by making them too costly to employ. Viral AI applications shape the dominant way of thinking and cultural sense making. This disrupts (non-)digital ecologies of knowledge and culture, at minimum leading to conceptual disruption and, at maximum, to conceptual loss, as happened in the context of historical colonialism. Historical colonialism, through physical violence and domination, was able to impose forms of conceptual adjustment and linguistic oppression.^12^

In response to these injustices and their dominative consequences, we argue that regional perspectives should inform the global stage of AI regulation. This form of “pedagogy of the periphery” is to be implemented by an institution for AI governance that adequately accounts for international differences in social groups without implying the reduction of other forms of knowledge and different kinds of knowers. It should be aware that those around the globe are disadvantaged in international forums for multiple reasons, including how sociohistorical factors have disrupted the concepts they appeal to in moral reasoning and its foundation. Therefore, recognizing the UN as a suitable source institution requires it to consider how AI has caused and will further engender epistemic injustices in the global South and address these harms.^3^

Before further strutting this argumentation with two principles in the later sections, it is worthwhile to become better acquainted with (1) cultural imposition and (2) hermeneutical injustice.

(1) How could non-inclusive global AI regulation become the root of cultural imposition on the global South? Cultural imposition has been a prominent feature of historical colonialism. Daniel Butt aptly describes the contours of this form of injustice: it “involved an attempt to impose the colonial power’s culture and customs onto the colonized, whether as a result of a belief in the racial and cultural superiority of the colonizing power; an evangelical desire to spread particular religions or cultural practices; or as a mechanism for establishing and consolidating political control.”^13^ We will not argue directly that exporting legislative models onto the global South constitutes a form of colonialism *tout court*. Still, we acknowledge that the comparison with colonialism is an insightful metaphor that discloses relevant similarities between the sociopolitical reality of AI and historical colonialism.^8^ We want to call attention to the strong resemblance between the colonial practice of cultural imposition and the export of the legislation, foreign technologies, and platforms that private and public life increasingly depend upon and revolve around. “Big Tech” does not hail from the global South, but its power over everyday processes is increasing. Likewise, Big Tech’s technologies incorporate capabilities, barriers, possibilities, and prohibitions created from a Western perspective that does not necessarily align with the diverse perspectives of the global South’s subregions—think of the Ubuntu morality from Sub-Saharan Africa^14^ and the decolonial perspectives from South America.^15^ Thus, a new wave of cultural value imposition is under way that proceeds via AI technologies: large language models or chatbots that were trained mainly on the Western Internet and other sources, leading to a Western “view from nowhere”^16^ interacting with inhabitants of the global South.

As such, Western values (which are not wrong per se) will permeate the global South from a new avenue: via human-AI interaction. An applied and intersectional example of this is found in work by African scholars:

Gendered chatbots in communities in Africa […] could introduce and, in some cases, further complicate gender relations in a way and manner akin to the impact of colonialism on indigenous women’s rights, which were eroded in colonial and postcolonial societies. […] Already, several studies have highlighted the many ways technology today is a tool of neocolonialism. This term refers to the indirect control and exploitation of a region or people by a more assertive foreign entity reminiscent of colonial extractivist activities. AI-powered chatbots could, therefore, introduce and impose new forms of gendered expectations upon women.^17^

As a countermeasure, calls for participatory forms of AI that would enable forms of connection with local values and socioeconomic needs have increased, in terms of both citizen participation and algorithm design.^18^^,^^19^ However, these calls have remained fragmented and will inevitably fall short of mustering global counterpower to Big Tech’s “hegemony” in AI capabilities if they are not united in a pluralistic legal embedding. In a similar vein, as one of us has emphasized elsewhere following Rachel Adams, calls for “decolonizing” AI—undoing it of Western universalism, so to say—require local targets integrated into global institutions, lest they fall short of the radical impetus of decolonial theory’s conception of social change to regions that are peripherical in terms of AI capabilities (e.g., Latin America or Sub-Saharan Africa).^7^^,^^20^ With current theoretical countermeasures being that difficult to realize, one can only grimly look at the prospective consequences of dissolving Western culture (values such as individualism, competition, progress, and norms) through digital interfaces and forms of social AI into the culture of the global South. Again, the harm here does not lie in the nature of Western values. Instead, the harm lies in the invasive character of the imposition and how this undermines forms of expression and doing in non-Western deployment zones. As Mhlambi and Tiribelli explain, unrestrained deployment in this sense can only result in restricting people’s autonomy from the global South by the constraints placed upon their form of life by the foreign interference of rules and legislation not tailored to their context.^21^ Indigenous cultural particulars are worthy of defense because their preservation is required for a prosperous plurality of values to vindicate differences against a monolithic universalism. This requirement underpins our research’s direction: formulate institutional principles to embed local approaches for developing and regulating AI.

Another prominent form of disruption to unpack is hermeneutical injustice. Understanding this concept is crucial to fully grasp the implications of AI regulation and its potential impact on the global South.

(2) Hermeneutical injustice involves disqualifying persons in their capacity as knowers.^22^ According to Miranda Fricker, who first conceived of this type of injustice, it is “the injustice of having some significant area of one’s social experience obscured from collective understanding owing to hermeneutical marginalization.”^1^ Applied to AI, members of social groups’ validity as knowing subjects is not taken seriously. AI systems contribute to the hermeneutical marginalization that underpins the actual injustice of someone being hermeneutically “handicapped.” Correspondingly, hermeneutical injustice plays into already existing asymmetries and inequalities in epistemic standing and socially ascribed weight to experiences, e.g., man versus woman or white versus black.^1^

In the AI context, hermeneutical injustice is brought about in two ways, the first of which, similar to cultural imposition, is via the exportation of standards of epistemological justification accepted in the global North. Essentially, this variant aggravates hermeneutical marginalization, making hermeneutical injustice concerning AI legislation more likely to occur. Standards of justification are embedded in legislation from the EU, such as the AI Act, or from the US or China, that determines which technologies they ought to protect and promote and which they should prohibit and bar. Hermeneutical injustice through uncontextualized legislation and AI systems is the flip side of the problem of cultural imposition: living under the domination of foreign values and standards of knowledge justification disrupts local identities and ways of life, which are dignified in their own right. The hermeneutical injustice in the digital realm made possible by AI technologies will not only aggravate the existing prejudices against the cognitive capabilities and types of knowledge of non-whites and non-Westerners,^23^ it will also create new dehumanizing asymmetries as long as a non-Western ideal of knowledge is propagated implicitly and explicitly in social AI and its corresponding regulation.

Second, hermeneutical injustice proceeds in direct interaction with AI systems that present an epistemological perspective that differs from the user’s perspective as universally valid or beyond doubt. According to McQuillan, “the complexity and opacity of AI-driven interventions are inherent barriers to any independent effort at comparable sense-making”; these barriers come to “[overlay] already existing cultural and institutional systems of superiority,” i.e., of hermeneutical marginalization, which drives AI systems’ hermeneutical violence.^22^ As one of us writes elsewhere, AI systems such as “LLMs amass a *surplus* of credibility, creating a *deficit* amongst average and culturally non-Western users. You have to be quite sure of your facts when it’s you against a personification of the Internet’s knowledge.”^8^ As the work of Yarden Katz and Muldoon and Wu illustrates, the Western biases involved with AI are caused by a training regime on almost entirely Western datasets, such as ImageNet.^15^^,^^16^ As Stanford University’s 2023 Artificial Intelligence Index Report indicated, biases are aggravated as a model’s size increases.^24^

For example, hermeneutical injustice occurs once an (already marginalized) social group cannot interact genuinely successfully with an AI system, e.g.: (1) when nuances in one’s native language are unintelligible to the system; (2) when indigenous concepts about local ecologies of knowledge are not recognized; (3) when the extent to which the workings of the AI system are not transparent for a social group; and (4) when there is structural bias of some other form in the AI systems. The “key performance indicators” of ethical AI, such as transparency, explainability, accuracy, and human likeness, are relative to culturally specific epistemic particulars. As Emma Ruttkamp-Bloem argues, “there is a feedback loop specifically between testimonial injustice and representational harm and between hermeneutic injustice and allocation harm (especially in the latter case if allocation harm is also seen in terms of access to equal quality education)” such that “excluding Africa from global discussions specifically in AI, given the potential of data-driven AI for amplifying structural bias, unfairness and exclusion, does far more harm than simply ensuring AI technology stays in the hands of the North.”^25^ Suppose AI systems are not developed by and for the global South. In that case, these AI systems’ benefits regarding capability enhancement will not be equitably allocated within the global South. The experience of lives lived within the cultures of the global South will be mismatched with the epistemological vantage point of the AI systems to the extent that citizens of the global South are structurally less able to benefit from the usage of AI systems than their counterparts in the global North. As such, there is a challenge to overcome that is specifically related to the AI systems deployed in parts of the global South, such as Africa: “epistemic injustice and being at the receiving end of structurally biased and non-transparent AI systems.”^25^

In line with elucidating the origins of hermeneutical and cultural AI injustice in the global South, Okolo et al. emphasize the importance of AI development being “for Africans by Africans” to ensure that colonial cycles of extraction by Western entities and historical dependence on foreign aid do not impede what could be a viable pathway toward economic freedom.^6^ Eke et al. echo this as well: “the needs of the Global North are different from those of the Global South; as such, it goes without saying that the application of AI on the African continent may be different in terms of the problems it intends to solve and subsequent benefits the technology will have.”^26^ These AI injustices call for a conceptual approach different from the Western universalistic conception of AI ethics. Just as with other provinces of practical philosophy, AI requires tailoring to the contexts of the global South.

Furthermore, hermeneutical injustice due to AI systems applies not only to the global South but also to the marginalized groups contained in countries of the global North. Hermeneutical injustice is always contextually determined by the hermeneutical marginalization of specific social groups whose epistemic and interpretative capacities are disqualified, derogated, or devalued regarding particular capabilities. Hence, the problem is not geographically restricted to the global South; it also concerns the loci of the global South in the North and can occur to marginalized citizens of the global North.^27^ For example, in the South, it may resemble colonialism, whereas in the North, it results in discrimination and oppression (in the sense of inhibiting social groups’ forms of self-expression). For both forms, if the development and deployment of AI are regulated along the lines of market-based values tuned to China, the US, and the EU only, the contextual complexity of those loci will be negated.

Therefore, we argue that just like existential risk due to superintelligence and global disruption, cultural imposition and hermeneutical injustice in the digital realm must be globally recognized as significant risks intertwined with the current power asymmetry between North and South and the latter’s economic dependence on the former. If this is recognized as a problem to be solved by global legislation, an important step will have been made on the stage set by the GDC. Cooperation between countries can be sought to strengthen local digital economies and participatory approaches to development. Regardless of whether the GDC is the appropriate embedding, its image demonstrates the need for discussions and more consensus on how the global AI superpowers can jointly commit to regulating the digital realm. This is especially the case among critical economies as they take divergent approaches to protect their constituencies and manage the risks and opportunities they present. Consensual discussions are an antidote to these states’ aims to build digital empires that, with the ambiguous support of their digital giants, control the world’s economic and political decision making through AI.^28^

Given this problematic reality and this even more problematic prospect, we propose two abstract principles to address these matters: recognitive consensus on key substantive benefits and harms, and procedural consensus on global coordination and essential standards. We will return to these principles respectively and present them as the main findings of the paper. As a prospectus of the argument, international rules concerning AI based on consensual justification are necessary to avoid power asymmetries that have epistemic injustice ramifications for the countries that do not own the tech or regulations.

## Framing regulation in the digital realm

To sketch the background of the discussion about the principles for the GDC, we have to define the term “regulation” broadly as a societal effort to channel digital technologies in the public interest and encompassing governance. By framing the definition as such, we draw from Ryan Calo, who prefers it over the term “governance” by arguing that while “governance” is attractive because of its flexibility, it is too flexible in that, in its usage, it is unclear what is being governed and by whom.^29^ This approach fits the United Kingdom (UK) Department of Science, Innovation, and Technology’s working definition of digital regulation: “the range of regulatory tools that government, regulators, businesses, and other bodies use to manage the impact that digital technologies and activities can have on individuals, companies, the economy and society” (https://www.gov.uk/government/publications/digital-regulation-driving-growth-and-unlocking-innovation/digital-regulation-driving-growth-and-unlocking-innovation). These include norms, self-regulation, statutory codes of conduct, and rules in primary legislation. The Department uses these tools to promote outcomes the market cannot efficiently achieve. Non-regulatory tools can complement or provide alternatives to “traditional” regulation. This includes industry-led technical standards, which benefit from global technical expertise and best practices.

Specific calls for regulation are becoming louder each day given the accelerating “capabilities of Generative Artificial Intelligence (GenAI)—including large language models (LLM)—as well as systems using real-time geolocation data, facial recognition, and advanced cognitive processing.”^30^ However, the international law of the digital realm currently needs to be more cohesive. Yet the diverse regulatory jurisdictions and regimes could run in tandem with and feed into the GDC to avoid a one-size-fits-all regulatory framework. Similarly, UN initiatives should coalesce around the Compact, like UNESCO’s AI Ethics Readiness Assessment (https://unesdoc.unesco.org/ark:/48223/pf0000381137). Apart from overlaps, gaps in the proposed regulatory regimes make it difficult for civil society and state governments to track the process, with the latter sometimes taking contradictory positions. While there are fears that the GDC could add new documents and contribute to further confusion, there is also the optimistic perspective that it will standardize governance, although it is not standard setting. Despite these challenges, some civil organizations have since filed recommendations intended for the first meeting of the High-Level Advisory Board on AI that they will propose to the UN General Assembly on this critical topic, such as the Center for AI and Digital Policy did (https://www.linkedin.com/feed/update/urn:li:activity:7113908196057976833/).

On the international level, consensus for regulating the digital realm is lacking, especially among critical economies such as the US and China, each with varied power, legitimacy, agency, and agenda. For instance, the UK has been taking the non-statutory approach currently under revision at the time of writing since the new labor government assumed power.^31^ The US takes a state-to-state-based approach to regulation while exhibiting a market-driven approach at the federal level. On the other hand, as Anu Bradford remarks, the Chinese and European regulatory models exhibit state-driven and rights-driven characteristics, and Singapore provides a hybrid case (https://www.linkedin.com/posts/anu-bradford-5036309_ai-singapore-us-activity-7108281438055088128-hXk4/?trk=public_profile_like_view). Partly driven by the geopolitical rivalry between the US and its allies on the one hand and China, Russia, and their allies on the other hand, this divergence is rooted in the antecedent jurisdictions that shaped the Internet’s early development, which is now embedded in the international, regional, and national Internet governance regimes. The US group prefers a more “open” Internet and a tightly restricted role for governments (Internet freedom). Conversely, the China-Russia grouping prefers a more centralized, state-led form of governance under the auspices of the UN (digital sovereignty).^32^^,^^33^^,^^34^ Therefore, critical attention must be devoted to Internet governance in the governance of emerging technologies such as AI.

Whatever form regulation takes, there appears to be an agreement that, ultimately, different nations will have different regulatory paths for AI worldwide; nevertheless, there should be an international consensus on broad principles on AI and global data flows. Dragoş Tudorache, a member of the European Parliament, stated that while there will ultimately be different regulatory paths for AI worldwide, interoperability between the regulatory regimes of democratic countries will be necessary.^35^ Similarly, there is a lot of talk on cooperation and policy collaboration, which was encouraged by the establishment of the US-EU Trade and Technology Council. Fissures in the US and EU are beginning to emerge with doctrinally different approaches to cross-border data sharing. While both blocks have passed measures to regulate international data transfers, their reasons differ. The EU couched its data transfer restrictions to protect Europeans’ privacy. However, its concerns over national security radiated through clash after clash with the US Government (over data from Passenger Name Records, the Society for Worldwide Interbank Financial Transfers, and the Snowden revelations per Schrems I and Schrems II). Oddly, the EU seemed to single out data transfers to the US while largely ignoring transfers to far more troubling destinations such as China and Russia. In contrast, in the Executive Order and Advance Notice of Proposed Rulemaking, the US Government explicitly focuses on national security—not privacy—while expressly supporting continuous data flows to democracies that adhere to the Organization for Economic Cooperation and Development (OECD) principles, including “all EU member states.”^36^ However, there is still doubt as to whether this will close the approach divergence, especially as the interoperability and standardization seem to omit China and the group of like-minded non-WEIRD countries.

Despite these doubts, there might be a shift in the future. Based on their current legislative activity and wider global influence, a review by Ernst & Young (on the varying approaches to AI regulation in Canada, China, the EU, Japan, Korea, Singapore, the UK, and the US) identifies five significant regulatory trends:^30^

(1) The OECD principles for AI, endorsed by the G20, serve as a global benchmark for AI, guiding policymakers
(2) Policymakers are adopting a risk-based approach to AI
(3) Policymakers are considering sector-specific aspects of AI oversight
(4) Regulators are increasingly considering how AI impacts other policy areas (e.g., data, cyber security, and digital content flows)
(5) Regulators are increasingly using sandboxes to enable the responsible testing of AI innovations^37^

What is salient about these points, at least *prima facie*, is that the OECD principles have taken on a global role that was only possible because of a lack of better initiatives. Its benchmarking has contributed to adopting approaches to countering risk and having adequate precautions. For example, the US Executive Order expressly supports continuous data flows to democracies that adhere to the OECD principles. At the same time, civil society, especially international NGOs and the UN, insist that international human rights norms and standards should be the “north star.”^38^ We argue that one act on these developments could be for regulation to prioritize the reinforcement of the legitimacy and relevance of existing international human rights frameworks and applying existing norms (as fleshed out by the work of treaty bodies, special rapporteurs, and so forth) to issues and harms arising from digitalization. Likewise, there is also a need to explore regulatory mechanisms and develop a comprehensive taxonomy that effectively categorizes these models, assessing their transferability across diverse global contexts and always ensuring alignment with the international human rights framework. One area that presents low-hanging fruits is to evaluate how the OECD principles could reinforce the GDC, as the tenets already draw broad consensus among several nations, including those in the global South.

## Main approaches to digital regulation

Alongside the efforts and the accompanying skepticism to craft a single global regulatory regime, several countries are paving diverse regulatory paths in response to the unique opportunities and threats presented by AI to their values, taking into account their history of rule making.^39^ Elaborating on the previously provided background sketch of the state of regulation in the digital realm, this section engages with the three primary strategies in digital regulation: the strategies of the EU, the US, and China.

The European Commission initially adopted a soft-law approach by publishing its non-binding 2019 Ethics Guidelines for Trustworthy AI and Policy and Investment recommendations (https://digital-strategy.ec.europa.eu/en/library/ethics-guidelines-trustworthy-ai). It has since shifted toward a legislative process, calling for the adoption of harmonized rules for developing, selling, and using AI systems. China already has AI regulations that balance innovation and social control in industry. While regulation is essential and presents a potential for more oversight, some firms such as OpenAI that have publicly called for more oversight have resisted some of the EU’s proposed controls and prefer international guidance bodies and voluntary commitments.^39^ The White House’s voluntary commitment to these companies presents an essential first step in building and using safe, secure, and trustworthy AI.^40^ Despite some form of consensus on the need for oversight over AI, there is no explicit agreement on its risks and benefits, how such administration should look, to what degree regulation is explicitly needed for AI, and whether existing laws might already address some of its risks as the technology is constantly evolving.

The differences between the global North and the global South in who “makes” and who “takes” legislation are more than arbitrary here and should be adequately historicized. The chasm in terms of legislation has not emerged out of the blue but is instead built upon a colonial dependency structure that continues until this day.^41^ More importantly for the topic of legislation, colonialism entailed a “conceptual adjustment program” that resulted, among others, in the destruction of rule-making concepts that were valued in colonized cultures.^42^ The outsourcing of legal concepts and concrete legislation from North to South can, as such, at least be approached in two epistemically unjust but co-extensive ways (as argued above): the cultural imposition of concepts and norms, which may or may not be in continuity with the colonial wrongs of the past that implied the same schema of exchange, and forms of hermeneutical injustice, as wrongs done to people in their capacity as knowers,^1^ in the way the imposed norms or routes of legislation may pose as universal, thereby disrespecting the legislation takers’ needs and standards of justification. This brief exposition prepares us for the next section, which shows that the global superpowers have vital doctrines upon which they base AI regulation that do not take the countries of the global South into account.

### Digital sovereignty and fundamental rights: The EU

In 2021, the European Commission proposed the AI Act to regulate AI. The AI Act passed on December 9, 2023 and will be operational in April 2026. The EU is the first-ever bloc to propose comprehensive AI regulations that seek to protect its sovereignty as regulatory power, strategic autonomy, and the ability to act in the digital sphere without being restricted to an undesired extent by external dependencies. Far from being normatively irrelevant, Europe has become, through the “Brussels Effect,” a “global regulatory hegemon unmatched by its geopolitical rivals.”^43^ While EU policymakers want the EU to become a regulatory superpower in AI, there are questions about whether this will succeed. Some believe that while parts of the AI regulation will most likely not diffuse, others are poised to have a global impact.^44^ The OECD, for example, criticizes some of the Act’s value terms, which may hinder its ability to protect the rights of EU citizens.^45^

In June 2023, the European Parliament adopted its proposed amendments to the draft EU AI Act to incorporate universal ethical principles and human rights as ultimate benchmarks for assessing the social acceptability of AI systems developed and deployed inside and outside the EU. The proposed amendments significantly reshaped the European Commission’s proposal by fine-tuning and expanding the scope of the draft EU AI Act, its risk-mitigation requirements, and its governance mechanism. The EU AI policies and regulations are both risk based and forward looking. In addition to the AI Act, the Digital Services Act (DSA) creates a safer digital space in which the fundamental rights of all users of digital services are protected. The Digital Market Act (DMA) establishes a level playing field to foster innovation, growth, and competitiveness in the European single market and globally. At the same time, they both form a single set of rules that apply across the whole EU.

At the same time, the proposed EU AI Act ensures safety and fundamental rights by imposing most of the responsibilities and obligations on “providers.” Unlike the General Data Protection Regulation (GDPR), a rights-based framework, the AI Act is based on product liability law (https://artificialintelligenceact.eu/the-act/). The proposed AI Act is also highly precautionary. It categorizes AI tools based on potential risks to ensure systems are safe, effective, privacy compliant, transparent, explainable to users, and non-discriminatory.^39^ Regarding Article 59 of the proposed EU AI Act, each member state must designate an authority responsible for implementing and applying AI regulation. For example, Spain recently created the Spanish Agency for the Supervision of Artificial Intelligence to guide the country’s AI ecosystem actively.

Since the EU rules are made at the EU parliament in Brussels, the belief that other states would voluntarily take up European digital regulation or model their regulations has, after Anu Bradford, become called the “de jure Brussels Effect”.^46^ Bradford highlights that the EU today: “promulgates regulations that influence which products are built and how business is conducted, not just in Europe but everywhere in the world.”^46^ In this sense, the major European legislations on AI and the digital realm, like the GDPR, DSA, DMA, and AI Act, are not restricted to Europe alone. Their formulation has an impact that ripples worldwide, reinforcing the legislative hegemony already in place. Countries in the global South must either comply or choose to do no bartering. This is not to say that these acts are without sound effects. Instead, it is the schema of imposition that is criticized. The countries in the global South cannot engage in self-determination in this respect, as the legislation is exported from the metropole they still depend on to the periphery where they remain enclosed.

### The markets-driven approach: The US

Like the UK, the US is taking a non-statutory approach to regulation. It recently confirmed it will continue with a principles- and context-based approach to AI regulation that avoids blanket rules that apply to all AI technologies regardless of their use case. The principles set by the UK Government are to be addressed by regulators through adjustments to existing laws (e.g., privacy, online safety, competition laws).^47^ While many leading AI firms are based there, a hands-off approach to regulation is under review.^48^ There has been an “appearance of activity” in the US.^28^ Silicon Valley technologists have generally viewed regulation as an obstacle to innovation. Efforts by Congress or the White House to regulate AI may face Supreme Court challenges under the central questions doctrine, which requires agencies to have explicit congressional authorization for significant decisions. Doing so would allow Congress to delegate AI-related tasks to expert agencies for rule-making and enforcement. In October 2023, President Biden passed an executive order outlining federal regulations on AI, for example, testing requirements to ensure systems cannot be used to create biological or nuclear weapons (and federal government reporting) (https://www.whitehouse.gov/briefing-room/statements-releases/2023/10/30/fact-sheet-president-biden-issues-executive-order-on-safe-secure-and-trustworthy-artificial-intelligence/). However, these will likely face legal and political challenges.^48^ The lack of a firm position has seen different states taking a piecemeal approach; for instance, the New York Senate and California were the first states to pass AI-specific legislation that balanced innovation and risk avoidance (https://www.gov.ca.gov/wp-content/uploads/2023/09/AI-EO-No.12-_-GGN-Signed.pdf). More recently, this “Californication” of US AI legislation continued when the State of California introduced another bill on AI that “requires companies to test the most powerful AI models before releasing them (i.e., test tools for ‘unsafe’ behavior, institute hacking protections, and develop tech in a way that it can be shut down completely) and to disclose the testing protocols and safety guardrails they have implemented.”^49^ If efforts like this keep succeeding, they will have a significant impact either on where the objects of legislation (i.e., Big Tech and their products) are situated or on the global market as a whole because ensured compliance would introduce and place constraints on many affiliated (sub)markets as well. Although the EU and US strategies share a conceptual alignment on a risk-based approach, the US prefers to focus on risks regarding how models will be used while the EU focuses on how they are also developed.

However, what about the US’s reluctance to regulate in the long term? Frequent calls of CEOs to testify in Congress put internal pressure on the affected companies themselves but do not contribute directly to obtaining global consensus. Again, we can ask: what are the effects on the global South? This is difficult to answer, but one thing is sure: these countries are more of a battleground than a stakeholder in the US AI legislation. However, suppose we take a look at its legislative history. In that case, the US’s reluctance to constrain the power of companies whose algorithms constitute the public sphere might create an atmosphere of suspicion and raise questions about the legitimacy of US and allied governments’ digital rights and freedom agenda in the same way the Snowden disclosures did on Internet freedom. After the Snowden disclosures, the legitimacy and credibility of the “Internet freedom” camp were considerably weakened, at which point the renewed concerns about the future of cyberspace governance began. There is a risk that the Internet and the digital realm will be permanently fractured, thus reversing the years invested in building the World Wide Web. Just as in the aftermath of Snowden, policymakers concerned about being cast as a pariah or an infringer of human rights will now have a convenient excuse supported by European and other government practices preaching “technological sovereignty.” Normalizing domestic information controls under the guise of technological sovereignty could have international repercussions through standard setting and “surveillance-by-design” infrastructure development projects. While still less ad hoc than the UK’s principle-based approach, the US’s fragmented state-by-state-billing approach to AI governance might leave too much interpretative room and time for AI harms to be adequately pre-empted.

### Aversion of social risk: China and Singapore

In China, the government is trying to balance innovation with retention of its tight control over corporations and free speech. With its aspiration to become the world leader in AI by 2030, monetize AI into a trillion-yuan industry, and emerge as the driving force in defining ethical norms and standards for AI, China’s AI strategy is worth examining.^50^ China’s strategic focus on AI includes international competition, economic development, and social and moral governance. Recent attempts to define ethical principles include the Beijing AI Principles and those established by China’s Ministry of Science and Technology. Understanding the areas where China considers AI to present opportunities is crucial to understanding China’s strategic focus. At the international level, in November 2023 China extended its Belt and Road Initiative through the Road Forum for International Cooperation, which might see more global Southern countries adopting the Chinese regulation model.

It is essential to evaluate China’s normative influence, if any, on its neighbors. This evaluation is necessary in light of the new role China is now playing as a global AI superpower that was not many years ago part of the “wretched of the Earth.” China’s model is focused not on enabling liberal AI markets, like the US approach, or securing internal markets’ safeties only, like the EU approach, but rather on obtaining sovereignty and the desired imperialistic control of peripheral countries that have come to have China as a metropole. As such, the Chinese type of regulation can hardly be mirrored, apart from in the form of being China’s accomplice. China’s position can partly be seen in Singapore, which presents an interesting example of balancing geopolitical tensions by embodying the state and market-driven models. While most Association of Southeast Asian Nations (ASEAN) countries have been known for mirroring Chinese Internet sovereignty norms, they have relied on the draft ASEAN “guide to AI ethics and governance.” In contrast to that voluntary guide, during the Davos Forum, the Philippines announced a plan to formulate a Southeast Asian regulatory framework to set rules on AI based on the country’s legislation, to be presented when it chairs the ASEAN in 2026. The challenge will be tackling ASEAN’s widely divergent legal systems, cultural values, and rules that govern censorship, intellectual property, misinformation, social media, and Internet use. Still, the hope is that member countries collaborate to develop comprehensive guidelines that consider the national context (10 countries and almost 700 million people) while ensuring consistency, with a focus on generative AI.

The way toward a global consensus is still opaque, but the West is mimicking the Chinese approach in their increasing appeals to consolidations of digital sovereignty. There are some similarities between the Chinese guidelines and those in the West. However, finding a common language with China will be difficult given differing views on privacy and societal control (i.e., how AI is used in law enforcement and the philosophy of protecting individual people versus national security measures). Chinese Premier Li Qiang noted that China wants to “step up communication and cooperation with all parties” to improve global AI governance.^51^ Could this signal that a form of consensus is in sight?

## Two critical principles for the GDC

### Preliminaries for the candidate global legislation principles

While there is skepticism about the possibility of a single global digital regulatory regime, a loose consensus on the need for shared regulation is emerging at the intergovernmental level. The November 2023 Bletchley Declaration during the UK AI Safety Summit is one step closer to the consensus, as it supports “an internationally inclusive network of scientific research on frontier AI safety that encompasses and complements existing and new multilateral, plurilateral and bilateral collaboration, including through existing international forums and other relevant initiatives” (https://www.gov.uk/government/publications/ai-safety-summit-2023-the-bletchley-declaration/the-bletchley-declaration-by-countries-attending-the-ai-safety-summit-1-2-november-2023).^52^ However, there are doubts about whether political leaders can develop the necessary policies when they know so little about AI, whose effective policy depends on a better understanding of the technology and its business models.^53^ The international caution is also reflected at some national levels, an interesting example of which is the Māori perspective (https://www.linkedin.com/pulse/artificial-intelligence-regulation-from-m%2525C4%252581ori-taiuru-jp-minstd-ga2zc/?trackingId=ENtKFVxlQsKDaHdr%2BuIBZA%3D%3D), which tends toward “caution in developing any substantial new law without first understanding the complex interaction of existing legal regimes. Before acting, it is essential to understand how these regimes apply to factual scenarios as they arise. Where new law is necessary, it will likely be a nuanced amendment to existing regulation. For now, existing legislation should be allowed to deal with harms from synthetic media technologies as they arise.”^54^

Despite the doubts, some AI thought leaders in the West believe that the West should engage with China’s regulators and experts, as their experience developing and implementing AI law and policy would be in the best interests of Western regulators as they work to set their policies.^55^ Further, for political actors to regulate AI effectively, AI developers, mainly those developing powerful generative models, should be less opaque and more transparent in giving data access for researchers—“a critical component for understanding and regulating AI effectively. It’s time for transparent standards and open data to lead the way to the robust, relevant policy that addresses many AI risks.”^56^ The researchers can, in turn, develop tutorials for policymakers.

Power over the global public sphere should never be handed over to corporate or private power, even though some note that any efforts by governments to regulate AI are meaningless and destined to fail. Such a conclusion is based on the observation that the real power resides in Silicon Valley and other technology hubs where AI is being developed, and there is no point in governments picking a fight that they will lose. Regardless of the contrary arguments, the pro-regulation sentiments are substantial; for example, Anu Bradford, a leading regulatory scholar at Columbia University, thinks that governments must insist on their role as the primary rule-makers working closely with AI developers to preserve the potential benefits of AI as key to regulating any fast-evolving, multi-faceted technology. She notes that close consultation with tech companies is one thing, while simply handing over governance to the private sector is another.^28^^,^^46^ Even though there are polarized opinions between those who favor the institutional and those for the free and horizontal approaches, in between the regulation and non-regulation camps there is consensus on the need for some form of regulation underpinned by procedural and substantive principles on fundamental shared issues. At the same time, the democratic countries have continued to take measures to address the “Huawei problem”—the term used for the awkward position of non-Chinese states that they (fully) depend on Huawei, as the largest 5G mobile network equipment supplier, for parts of their digital infrastructure—casting skepticism over the possibility of any global consensus involving Russia and China, as it might lead to regulatory capture.

To address the Huawei problem and the fears of strengthening the role of the UN through which China and Russia have traditionally lobbied smaller countries through their normative influence, we argue that convergence on the global level is not expected based on the convergence in appeals to digital sovereignty. Why? Digital sovereignty conflicts with the underlying will to expand and the states’ divergent philosophies underlying their approaches.^57^ The US, EU, and China are concerned with global influence and are not just safeguarding separate insular digital spheres. Therefore, the boundaries of their desired sovereign spaces overlap. Cooperation may be sought to prevent tensions from skyrocketing, but the economic interests are too heavy. Still, what would the consensus of the superpowers mean for the global South? The global South looks at this geopolitical stage from the sideline or experiences it from the inside out as a contingent battleground for digital influences and resources. One is tempted to make a grim comparison with the 1884 Berlin Conference. This partitioning of Africa among the colonial superpowers of the time sealed Africa’s future for a long time. Whatever the global equilibria for AI regulation in the future, Africa, among other smaller markets of the global South, is not one of the designers right now. This situation is suboptimal for this market and others in the long run and is a source of foreign interference that cannot be evaded and will have far-reaching influences on the shape of future development and deployment of AI in the global South.^58^

What the consensus of the superpowers would mean for the global South is still debatable. However, the world cannot sit idly by and allow the history of colonization to repeat itself. It can draw inspiration from how the world has reached a political consensus on the existential challenges of the past. Think, for example, of the 1948 Universal Declaration of Human Rights. Although the result was not realized regarding national legal rights, it subsequently led to several binding covenants. As we suggested at the start of this paper, the UN GDC shows the possibility of a worldwide framework for AI regulation (https://www.un.org/techenvoy/global-digital-compact; https://undocs.org/en/A/RES/75/1). At a normative level, such an arrangement would ensure the desired degree of universalization and formalization of the AI and data principles and laws. AI rules must subsequently be judged according to legal validity and not subjected to normative inquiry.

Yet is the GDC itself an adequate reflection of an ideal institutional embedding for consensual discussions on AI? As a product of a structurally unjust global society, probably not. But, whether or not that is the case, the Compact nevertheless demonstrates the need for discussions and more consensus on how the global AI superpowers can jointly commit to regulating the digital realm. However, returning to the dangers of cultural imposition and hermeneutical injustice that we named earlier, what principles should the GDC embody to ensure it does not again lead to a Western normative dominance that once again does not take the global South seriously? What is the ideal conceptual underpinning for such an approach?

The remainder of the paper aims to explore and judge the potential of core shared principles for AI governance and why they matter if consensually justified and agreed to, then argues for their conceptual justification. Therefore, our reflections here are conceptual, normative, and anticipatory but speak to action in a few ways (https://www.hrw.org/news/2023/10/05/russia-china-unfit-uns-top-rights-body).

At an empirical policy level, core principles matter when they absolve current tensions created by different paths to regulation, the disagreement on issues including risk with two camps on immediate as compared to the apocalypse, lip service being given to cross-border data flows and dangers posed by big compute, and finally procedural issues relating to global standardization and coordination. The procedural issues are rooted in the antecedent of Internet governance, which has always pitted those countries that favor a multi-stakeholder approach against those with a state-centered approach to regulation. On a conceptual level, they matter because of the risks of causing cultural imposition and hermeneutical injustice, but how would those injustices be causally related to the domination of the global South in terms of AI governance? (1) The global South lacks adequate regulatory protections, for example, unsupervised and unregulated collection and use, and (2) none of the governance models surveyed has the historical context of the global South and its subregional specificities in mind, for example, collective approaches. The most significant impact of the lack of regulation is primarily on historically disadvantaged and marginalized groups, and the result will be the amplification of these “digital” inequalities worldwide.

Suppose we continue forward without questioning the trajectory. In that case, the global South is only at power to import legislation rather than craft its own because of the domination of the digital market by the US in terms of platform economies and software, China in terms of infrastructure, and Europe in terms of legislation. We should thus expect to see increased global inequality alongside local economic disruption and social unrest due to the unregulated deployment of new technologies that citizens and states are not educated to deal with. Suppose Africa continues to be the theater of geopolitical rivalries. In that case, further political instability might result, with the technologically disadvantaged and under-represented faring the worst. This is a legitimate concern but, that being said, the problem that China and Russia are unfit to have vital roles in participation in an organization like the UN is being raised across the board because of their structural human rights violations. China’s use of AI in its social credit system and surveillance technologies is a standard-setting violation of human rights through digital domination. With AI’s impact growing, it is no longer a market-share issue but a prominent human rights issue. Therefore, any fear of global consensus failing to be attained because some of the UN’s participants do not value human rights is unfortunately grounded.

Based upon the previously discussed preliminaries, we argue that the GDC should be based on two fundamental principles that resolve substantial issues and procedural matters in a complementary manner to ensure non-arbitrariness in the choice of the matter. The first principle is “recognitive consensus on key substantive benefits and harms.” In short, this principle holds that some global benefits and harms require a pluralist recognition and distribution, whereas a Western universalist policy does not dominate both. The second principle is “procedural consensus on global coordination and essential standards.” At its core, it entails that an institutionally guided and inclusive process should underpin global coordination and the creation of standards for AI safety and deployment. The following proposes and discusses the two principles in turn.

### Principle 1: Recognitive consensus on key substantive benefits and harms

The first principle is identifying and recognizing shared issues and working toward a consensus. Why is this principle necessary? Some questions are about translating broadly shared issues to particular applications. What are the expected cross-border benefits and harms? How should the problem be defined such that the gap between the different understandings is narrow? This principle posits that the required consensus is based on a pluralist consensus of states that global regulation should be broad and try to give little detail to avoid the pitfall of the imposition of a one-size-fits-all approach. Different issues cannot be solved by a direct one-size-fits-all approach. A first example is the issue of cross-border data flows and the “upstream” of controlling computing power, given that controls such as physical limits on chip-to-chip networking or cryptographic technology allow for remote disabling of AI chips in extreme circumstances.^59^ Second, clarity has been gained regarding the gravity of the environmental costs of AI, both in terms of energy consumption, which requires fossil fuels for production, and the amount of water consumed.^60^ Third, how could AI be used in a manner that respects the particular social group’s data rights, corresponding to claims from the global South, to resist the Western individual-centric focus that is imposed on them?

This principle of recognitive consensus is critical now, given the recent shift by the US Trade Representative when it abandoned (https://ustr.gov/about-us/policy-offices/press-office/press-releases/2023/october/ustr-statement-wto-e-commerce-negotiation) its long-standing demand for World Trade Organization provisions to protect cross-border data flows and prevent forced data localization, which some consider a threat to “the very survival of the open internet, with all the knowledge-sharing, global collaboration, and cross-border commerce that it enables.”^61^ Each region’s particularities should run in tandem with the global discussion informing the regulatory frameworks of different powers to the possibility of a worldwide framework for AI regulation, as shown in the GDC, which outlined shared principles for an open, free, and secure digital future for all: (1) including digital connectivity; (2) avoiding Internet fragmentation; (3) providing people with options as to how their data are used and application of human rights online; and (4) promoting a trustworthy Internet by introducing accountability criteria for discrimination and misleading content.

While the Compact should be forward looking in pre-empting future harms and addressing immediate harms, and there is consensus on the need for some regulation, the threat to consensus comes from the different understandings of AI risks. The apocalyptic camp thinks that AI poses a severe threat to humanity.^53^ This is illustrated by the plethora of injunctions and warnings issued by leading AI think tanks regarding so-called existential risks (https://futureoflife.org/open-letter/pause-giant-ai-experiments/). Contrarily, some, for example Timnit Gebru, think such fears are exaggerated. Consider her critical appraisal of the global conversation:

“That conversation ascribes agency to a tool rather than the humans building the tool,” she says. “That means you can abdicate responsibility: ‘It’s not me that’s the problem. It’s the tool. It’s super-powerful. We don’t know what it’s going to do.’ Well, no – it’s you that’s the problem. You’re building something with specific characteristics for your profit. That’s highly distracting and takes the attention away from actual harm and things we need to do. Right now.”^62^

Her views are echoed by the UK House of Lords, which recently said the apocalyptic concerns about threats to human existence are exaggerated and must not distract policymakers from responding to more immediate issues. Near-term security risks include cyber attacks, child sexual exploitation material, terrorist content, and disinformation. The Committee says that catastrophic risks are less likely but cannot be ruled out, noting the possibility of a rapid and uncontrollable proliferation of dangerous capabilities and the lack of early-warning indicators. However, as mentioned earlier, neither the utopia/dystopia distinction nor the focus on immediate harms adequately conceptualizes the prospect of AI harms for the global South. While existential risks should not be downplayed entirely, one should question the framing of deeming this risk the most important. As one of us argues elsewhere, the existential risk framing of wealthy parties in the global North “intensif[ies] capital investments into the digital economy with colonial injustices as collaterals” all the while “minimizing future suffering while remaining ignorant of actual suffering.”^7^ Therefore, one must remain conscious of whose livelihood is at stake in such a fear. The immediate consequences of affective disruption that, for example, Yuval Noah Harari warns of, and that are already possible—as pre-LLM misinformation wars in Kenya, among other places, already show—would be much more significant in the global South because technological counterforces, regulations, and infrastructures are less well developed than in the North.^63^^,^^64^ Both the utopia/dystopia and the immediate harm conceptualizations fall short of having global coverage because they remain rooted in Western societies, concerns, and fears. The cases of cultural imposition and hermeneutical injustice illustrate that both existential risks and immediate harms will be experienced differently in the global South, refracted as they are through the aperture of post-colonial power asymmetries. This makes clear that the GDC needs to spell out and balance the two types of risks, whereby it has to ensure that no fear is privileged because of the stakeholder behind it rather than because of the probability of risk and its consequences.

The resulting strength of this principle is that it avoids the pitfall of universalism by striving for a genuinely plural institutionalization of AI governance. It thereby provides starting points for regional legislation to latch onto. Concerning its realization, regions with robust rulemaking traditions, such as the EU, could provide the much-needed resources and expertise at the norms and standard-setting bodies such as the UN. Nevertheless, the UN should put measures in place to avoid value imposition like the AI Act already risks doing via its clause that ensures the global reach of its application. To do so, various UN offices should continue with their normative leaderships in a pluriform way, making room for more perspectives than that of just the “big three.” For instance, the UN Secretary-General should facilitate member states to negotiate the GDC. However, allowing countries to choose which values to align with might deepen the chasm between the countries that adopt competing approaches to digital regulation. Given the exposition we presented of the main approaches to AI regulation, liberal tendencies toward digital sovereignty are discernible in all parties’ strategies. A broad recognitive consensus can ensure the component of positive liberty present in this tendency, thereby enabling regional control over the development of AI industries and minimizing digital foreign interference. In contrast, compliance with a broad global framework for AI safety ameliorates the nationalizing and dictatorial possibilities enabled by completely sovereign governance of the digital realm. To make it concrete, for example affording the EU to play a role in guiding rulemaking, will ensure that it cannot be a player and a referee simultaneously, and this has the practical effect of restraining the Brussels Effect to comply with the international rules-based order that the EU itself cannot dominate. A shared AI governance and safety framework could also help constrain the current “digital cold war” between the US and China, benefiting geopolitical stability.

The consensual nature of the principle is necessary because there are, after all, general risks and problems shared by all countries that participate in the global digital economy, which are not only existential in type. The nuance with which the concept of risk should be approached here is that it should concern the globally shared issues relative to the views of all stakeholders. Views from the global South also need to be represented and recognized as valuable in their own right. This sounds like an essential public sphere requirement, but the digital dominance of the economic superpowers skews the conversation before it can even start. The penultimate section will discuss how this principle helps counter the forms of domination enabled by cultural imposition and hermeneutical injustice.

### Principle 2: Procedural consensus on global coordination and essential standards

The second principle is to have procedural consensus on the global coordination of AI governance and formulation of essential standards for AI legislation and safety approaches. International standardization and coordination are the keystones to consensus on distributing critical harms and benefits. If the potential wrongs go unrecognized, they could lead to the unfair distribution of the goods AI can potentially yield. What is needed to adequately tackle the wrongs of cultural imposition and hermeneutical injustice is an institutional embedding that is at the same time standard setting, flexible with regional extension and applications, and wields the power to enforce its standards or, at the very least, have a joint commitment to rebuke where the parties fail to fulfill their obligations.^65^ International standards should be flexible because they generate guardrails for local applications but leave room for contextual and cultural adjustments. This is a complex principle to realize, but it should not be deserted for that reason. With foundation models posing the most apparent threat of cultural imposition by the Westernized Internet’s values seeping into every one of its overhead applications, standards and best practices on an abstract level are needed for anti-colonial, locally beneficial, and nurturing forms of AI to be developed in the global South, unthreatened by foreign market dominance.

Despite the different regulatory paths already in sight in the global arena for the regulation of AI, states could agree on the interoperability between their regulatory regimes through adherence to shared standards.^35^ Standards for AI governance could play a mediating role here. Although the Compact will not cover every aspect of digital technology, standards for how duties to transparency are realized must be harmonized as much as possible to minimize overheads to businesses operating across jurisdictions. The EU could provide leadership here, as its member states are now preparing to adopt standards to comply with the EU AI Act. This is an aspect where the Brussels Effect, if not orchestrated solely for the EU’s benefit, could prove beneficial for the global South as well, as the AI Act, in contrast with the GDPR, is not only a set of definitions but also leaves room for standardization and best practices. “Only a concerted global effort can ensure that the Internet isn’t increasingly fragmented, insecure, and controlled by governments and corporations,” as Campbell and Adams write.^61^ Therefore, it is imperative that we, as a global community, exercise public control over private power in the realm of AI governance. This responsibility is not to be taken lightly, as it will shape the future of AI and its impact on society.

Global standardization and coordination are essential for creating adequate guardrails that extend beyond the EU. An international standardized approach can best be achieved through a multi-stakeholder approach premised on a clear division of labor between states, corporations, academia, and civil society. This approach is not new, as it reflects the current approach to Internet governance in mechanisms such as the Internet Corporation for Assigned Names and Numbers. Currently, the WEIRD countries prefer a more “open” Internet and a tightly restricted role for governments, in contrast to those countries that prefer a more centralized, state-led form of governance, preferably under the auspices of the UN. In our view, the limited role of the UN (especially the Office of the Envoy of Technology [OSET]) in coordinating the two principles we propose is compatible with the open multi-stakeholder approach. The current OSET mechanisms have allowed civil society to contribute to the UN Tech Envoy’s Five-Point Plan for AI Governance and the Universal Guidelines for AI as the basis for the global governance of AI. While further points of detail and even the principle of the mechanisms remain to be spelled out, inspiration can be drawn from historical approaches to governance and then adjusted. For example, in his recent article for Rand, Michael Vermeer identifies three key themes to technology governance: sustained consensus on norms for the technology, governance of physical and non-physical assets, and public-private partnerships in governance.^66^

Although the principles we propose sound merely aspirational, their critical importance is that existing mechanisms within and outside the UN are emerging that do not just acknowledge their need but also provide concrete entry points for recognitive consensus on benefits and harms and procedural consensus on standardization and coordination. The appropriate momentum is thus coming into being. Examples are the Council of Europe Convention and the UN’s “blueprint for AI.” Firstly, the Council of Europe treaty is about protecting human rights, democracy, and rule of law with respect to AI and “commits parties to collective action to manage AI products and protect the public from potential misuse” (https://www.gov.uk/government/news/uk-signs-first-international-treaty-addressing-risks-of-artificial-intelligence). If treaties such as this provide international inclusion and support over and above the EU-US axis, they can support the development of regionally specific responsible AI governance that still adheres to international agreements with respect to democratic and legal foundations. Secondly, according to Cameron Kerry, a leaked report from the UN’s High-Level Advisory Body indicates an impetus to be involved in realizing international AI governance.^67^ Kerry claims the involvement of the UN should be “as a facilitator rather than a policymaker” because “existing systems meet the initial draft’s goal of being agile, networked, and flexible.” We, however, beg to disagree and wish to point out the necessity of the incorporation of the recognitive and procedural principles on the UN level in a regulatory rather than facilitatory form. Contrary to Kerry, we want to emphasize that there is a lack of recognition of the stakes of UN members hailing from the global South. The principles we propose require binding or strongly steering mechanization at the UN level. As the long-term distribution of the benefits and burdens of AI is going to be decided in the coming struggle over dominance in the AI governance arena, recognition and consensual standardization hailing from this international authority are imperative. In line with what Pouget et al. report, the UN should strive for deep involvement with its member states on “a broad range of AI-related issues”; at the same time, we agree with them that no single institution can foster total agreement of AI’s risks and their governance, but remain convinced that there is a regulatory rather than advisory and facilitative role to play for institutions coordinated by the UN.^68^ After all, if achieving recognitive consensus and consensual standardization are not mandatory, achieving the potential benefits of these principles has become utopian rather than the implementation of the principles themselves.

## Discussion

Preliminarily, it is worthwhile describing the interrelation of the principles. The first principle precedes the second one. Recognizing common issues or essential uncommon particularities (Principle 1) must be present before these aspects can be incorporated into procedurally just coordination or standardization (Principle 2). Coordination of governance or AI safety standards regarding these topics is preceded by recognizing the issues that need to be governed and standardized.

Accordingly, the two principles work together. The central merit of the two principles is that they enable a precautionary countering of cultural imposition and hermeneutical justice. As expounded previously, cultural imposition results in forms of domination rooted in autonomy restrictions. Positing a broad consensual framework upfront in the GDC enables the incorporation of the necessary regional values and concerns regarding AI systems in the local application and institutionalization of AI legislation, particularly in the global South. On the other hand, hermeneutical injustice is rooted in an epistemic mismatch between (1) the exported legislation and AI systems and (2) the deployment context of the global South. By bringing the global South’s concerns to the fore via Principle 1 and developing regionally specific coordination and standards sensitive to this context via Principle 2, the occurrence of disruptions of epistemic particulars in interaction with AI systems is made less probable. Therefore, the interplay of Principles 1 and 2 addresses the three forms of domination related to cultural imposition and hermeneutical injustice, namely (1) digital domination, (2) the oppression of local AI development, and (3) the vitiation of cultural and epistemic conceptual resources. We now sketch the application of the principles to these three forms of choice set restriction.

(1) Digital domination is pre-empted because the regional specificity of coordination and standards enabled by both principles obstruct the export of foreign legislation, governance frameworks, and ethics benchmarks that hail from (falsely universal) cultural and hermeneutical standpoints.
(2) Local AI development is fostered rather than oppressed by the two principles because of the standard precautions taken against the recognized shared issues at the global level of the UN (Principle 1), while Principle 2 supports regional applications of AI technologies to allow them to be tailored to the cultural and epistemic particulars of the AI capabilities that are relevant in a specific context.
(3) Conceptual disruption via cultural and epistemic colonialism is also obstructed by the combination of the principles. This is because the global South, by Principle 1, will not be dominated by foreign conceptions of AI risk but will have furthered the global recognition of the dangers and challenges its communities envision, and, by Principle 2, will develop its own eudaimonic and communitarian vision of the AI capabilities that are relevant to foster, prohibit, and legislate. The corresponding consequence is that AI systems for and by the global South—not hijacked by foreign agendas—will be able to emerge.

However, our optimistic and still conceptual vision of adapting and implementing these principles leads to entry points for further discussion.

Firstly, what if a broad global consensus is not obtained and neither principle can be supported and implemented accordingly? We want to briefly note two alternative strategies that can be pursued to preserve the gist of the two principles but cannot do without the global recognition and institutionalization strived for by the GDC. The first alternative is fostering local AI capabilities. Apart from any global institutionalization, regionally significant AI capabilities can be identified and embedded into regional frameworks for AI governance. The development of those AI capabilities can be stimulated and regulated, leading to AI systems and practices tailored to the region’s needs in the global South better than any Western or Chinese off-the-shelf foundation model can be. Another route toward fostering AI capabilities is laying constraints on what can be done with non-indigenous AI in terms of algorithmic decision making and other high-risk AI applications. The second alternative is decolonizing AI. AI development and deployment contain traces of colonialism and, in some respects, converge with it in effects. In line with what both of us argue elsewhere, the “colonial” ecological, political, and epistemic barriers in AI research, regulation, and systems must be identified and “disenclosed,” i.e., undone, prevented, and repaired. The values of the historically oppressed and those newly oppressed by digital means must be recognized and broadly construed in AI. These forms of decolonization can be pursued as regional projects, local forms of resistance, and national goals, apart from any global recognitive or procedural consensus principles.^7^^,^^69^

Secondly, some would say that the issues posed by (generative) AI strongly differ from those posed by the data flows of cloud and mobile technologies that legislation—until the AI Act—has focused on. Hence, it is relevant to ask: do both principles adequately consider the distinctiveness of (generative) AI? To begin with, it is pertinent to note that there is a strong continuity between what is now called “generative AI” and what was up until 2015 called “Big Data.” The algorithmic processing and harvesting of vast amounts of data is one of the commonalities between the two. Current AI systems have advanced over other machine-learning applications in their practical applicability because of their generative capacities and the possibility to tailor them to diverse use cases. It is, however, mainly the new “high-risk” aspects of these AI systems that have, up until now, remained unregulated globally. Examples of such high-risk AI systems, as the EU AI Act names them, are systems related to financial, medical, or socio-bureaucratic decisions directly affecting persons because biases, malfunctions, and so forth could have racist, discriminatory, or simply far-reaching consequences for the persons subjected to the system. Bringing this back to the principles of recognitive and procedural consensus, one can see how they relate to precisely these semi-autonomous aspects of high-risk AI systems that are not involved in the cloud and mobile technologies per se. The distribution of the benefits and harms related to the cross-border deployment of high-risk AI systems is an issue where consensus is needed to recognize the regional particularities, needs, and wishes of all stakeholders involved (Principle 1). These regional particularities, needs, and wishes translate into cultural and epistemic resources differentially being affected by AI systems in the global South as compared to the global North. Therefore, a broad procedural consensus regarding coordination and standardization is necessary for emerging local and regional regulations to better address the differential effects on cultural and epistemic resources (Principle 2).

Thirdly, this paper could not address some directions for future research that remain notable. For example, the analysis provided here could be enriched by case studies concerning the deployment of AI systems in the global South to better chart the forms of cultural imposition and hermeneutical injustice and test the principles proposed here. Equally relevant to further exploration is the question which mechanisms would be most suitable to implement the candidate principles. Lastly, our normative argument can be subject to further evaluation. For example, one can ask: could other principles be discerned to supplement the antidote to the forms of domination enabled by cultural imposition and hermeneutical injustice? To conclude, our argumentation has opened up multiple avenues for further research and discussion.

## Conclusion

The GDC is a promising advent of a new wind blowing in global AI regulation. This paper has contributed to discussing two principles that could underpin such a compact and for it to effectively pre-empt cultural imposition and hermeneutical injustice concerning AI. Recognitive and procedural consensus on standardizing and coordinating approaches to shared cross-border harms and benefits will not only mitigate the global risk posed by AI systems. It also takes precautions against the conceptual and dominative differential disruptions that will disproportionately harm the global South. Cultural imposition and hermeneutical injustice are more disruptive in the South than in the North, as the cultures and epistemic resources of the former have historically already been marginalized in global rulemaking. A coordinated and standardized approach to AI harms and benefits will ensure that the worldwide consensus draws from regional particulars and that international rules on AI do not just reflect the values of cultures with a robust rule-making tradition such as the EU. Although the EU can rely on its regulatory capacities to shape the consensus through the UN in a multi-stakeholder and open forum, it minimizes the risk of Brussels imposing European values on the global South through the Brussels Effect. Finally, we discussed how the principles of recognitive and procedural consensus help prevent the three forms of domination related to cultural imposition and hermeneutical injustice, and we sketched possible alternative routes. Given the rapid development of approaches to AI governance and safety, time is of the essence for establishing globally inclusive AI governance.

## Acknowledgments

This work is part of the research program Ethics of Socially Disruptive Technologies, which is funded through the Gravitation program of the Dutch Ministry of Education, Culture, and Science and the Netherlands Organization for Scientific Research (10.13039/501100003246NWO grant number 024.004.031).

## Author contributions

Conceptualization, A.G. and W.J.T.M.; writing – original draft, A.G. and W.J.T.M.; writing – review & editing, W.J.T.M.; supervision, A.G.

## Declaration of interests

The authors declare no competing interests.

## References

[1] Fricker M. (2007).

[2] Gwagwa A., Kazim E., Hilliard A. (2022). The role of the African value of Ubuntu in global AI inclusion discourse: A normative ethics perspective. Patterns.

[3] Nickel P.J., Kudina O., van de Poel I. (2022). Moral Uncertainty in Technomoral Change: Bridging the Explanatory Gap. Perspect. Sci..

[4] Hopster J.K.G. (2024). Socially disruptive technologies and epistemic injustice. Ethics Inf. Technol..

[5] Gwagwa A., Townsend B. (2023). https://www.globalpolicyjournal.com/blog/10/05/2023/re-imagining-africas-sovereignty-digitally-interdependent-world.

[6] Okolo T., Aruleba K., Obaido G., Eke D.E., Wakunuma K., Akintoye S. (2023). Responsible AI in Africa.

[7] Mollema W.J.T. (2024). Decolonial AI as Disenclosure. Open J. Soc. Sci..

[8] Mollema W.J.T. (2024). https://studenttheses.uu.nl/handle/20.500.12932/47214.

[9] Pettit P. (2013).

[10] Aytaç U. (2024). Digital Domination: Social Media and Contestatory Democracy. Polit. Stud..

[11] Sahbaz U. (2019). Artificial Intelligence and the Risk of New Colonialism. Horizons.

[12] Wiredu K. (2002). Conceptual Decolonization as an Imperative in Contemporary African Philosophy: Some Personal Reflections. Rue Descartes.

[13] Butt D., LaFollete H. (2013). The International Encyclopaedia of Ethics.

[14] Ugar E.T. (2023). Filosofia Theoretica: Journal of African Philosophy, Culture and Religions.

[15] Muldoon J., Wu B.A. (2023). Artificial Intelligence in the Colonial Matrix of Power. Philos. Technol..

[16] Katz Y. (2020).

[17] Borokini F., Wakunuma K., Akintoye S., Eke D.E., Wakunuma K., Akintoye S. (2023). Responsible AI in Africa.

[18] Birhane A., Isaac W., Prabhakaran V., Diaz M., Elish M.C., Gabriel I., Mohamed S. (2022). Proceedings of the 2nd ACM Conference on Equity and Access in Algorithms, Mechanisms, and Optimization.

[19] Lambrechts W., Sinha S., Mosoetsa S. (2022). Colonization by Algorithms in the Fourth Industrial Revolution. IEEE Access.

[20] Adams R. (2021). Can Artificial Intelligence Be Decolonized?. Interdiscipl. Sci. Rev..

[21] Mhlambi S., Tiribelli S. (2023). Decolonizing AI Ethics: Relational Autonomy as a Means to Counter AI Harms. Topoi.

[22] McQuillan D. (2022).

[23] Santos B.S. (2014).

[24] Maslej N., Fattorini L., Brynjolfsson E., Etchemendy J., Ligett K., Lyons T., Manyika J., Ngo H., Niebles J.C., Parli V. (2023). The AI Index 2023 Annual Report. arXiv.

[25] Ruttkamp-Bloem E., Eke D.E., Wakunuma K., Akintoye S. (2023). Responsible AI in Africa.

[26] Eke D.E., Chintu S.S., Wakunuma K., Eke D.E., Wakunuma K., Akintoye S. (2023). Responsible AI in Africa.

[27] Arun C., Dubber M.D., Pasquale F., Das S. (2020). The Oxford Handbook of Ethics of AI.

[28] Bradford A. (2023). https://www.project-syndicate.org/onpoint/ai-regulation-us-eu-china-challenges-opportunities-by-anu-bradford-2023-09.

[29] Calo R. (2017). https://lawreview.law.ucdavis.edu/issues/51/2/Symposium/51-2_Calo.pdf.

[30] Ernst & Young (2024). https://www.ey.com/en_gl/ai/how-to-navigate-global-trends-in-artificial-intelligence-regulation.

[31] Gallo V., Nair S. (2024). https://www.deloitte.com/uk/en/Industries/financial-services/blogs/the-uks-framework-for-ai-regulation.html.

[32] Goldsmith J. (2018). https://knightcolumbia.org/content/failure-internet-freedom-.

[33] Barlow J.P. (1996). https://www.eff.org/de/cyberspace-independence.

[34] Weber V. (2019). Oxford Centre for Technology and Global Affairs Working Paper Series.

[35] Overly S., Tudorache D. (2024). https://politico-tech.simplecast.com/episodes/the-eus-ai-guy-has-some-advice-for-congress.

[36] Swire P., Sacks S. (2024). https://www.lawfaremedia.org/article/limiting-data-broker-sales-in-the-name-of-u.s.-national-security-questions-on-substance-and-messaging.

[37] Madiega T., Van De Pol A.L. (2022). https://www.europarl.europa.eu/RegData/etudes/BRIE/2022/733544/EPRS_BRI(2022)733544_EN.pdf.

[38] Kaye D. (2018). https://knightcolumbia.org/content/limits-supply-side-internet-freedom.

[39] Hutson M. (2023). Rules to keep AI in check: nations carve different paths for tech regulation. Nature.

[40] Gupta A. (2023). https://abhishek-gupta.ca/aci/blog/moving-the-needle-on-the-voluntary-ai-commitments-to-the-white-house.

[41] Mbembe A. (2017).

[42] Okeja U. (2022).

[43] Christakis T. (2020).

[44] Siegmann C., Anderljung M. (2023). https://montrealethics.ai/the-brussels-effect-and-ai-how-eu-regulation-will-impact-the-global-ai-market/.

[45] Franklin M., Tomei P., Gorman R. (2023). https://oecd.ai/en/wonk/eu-ai-act-manipulation-definitions.

[46] Bradford A. (2020).

[47] Mclean S., Battersby K., Bange V., Steele J., Harford K. (2024). https://www.connectontech.com/uk-government-confirms-principles-based-approach-to-regulating-ai/.

[48] Sanger D., Kang C. (2023). https://www.nytimes.com/2023/10/30/us/politics/biden-artificial-intelligence.html.

[49] De Vynck G., Zakrzewski C. (2024). https://www.washingtonpost.com/technology/2024/02/08/california-legislation-artificial-intelligence-regulation/.

[50] Roberts H., Cowls J., Morley J., Taddeo M., Wang V., Floridi L. (2021). The Chinese approach to artificial intelligence: an analysis of policy, ethics, and regulation. AI Soc..

[51] Petty M., Chang R. (2024). https://www.reuters.com/technology/philippines-propose-asean-ai-regulatory-framework-house-speaker-says-2024-01-17/.

[52] Ruggeri A. (2024). https://www.bbc.com/worklife/article/20240118-davos-2024-can-and-should-leaders-aim-to-regulate-ai-directly.

[53] Seal T., Bloomberg (2023). https://fortune.com/2023/11/02/elon-musk-ai-regulations-uk-prime-minister-sunak-ai-safety-summit/amp/.

[54] Barnes C., Barraclough T. (2019). https://www.lawfoundation.org.nz/wp-content/uploads/2019/05/2018_ILP_5_RESEARCH-REPORT_Perception-Inception-Report-EMBARGOED-TILL-21-May-2019.pdf.

[55] MacCarthy M. (2023). https://www.brookings.edu/articles/the-us-and-its-allies-should-engage-with-china-on-ai-law-and-policy/.

[56] Schaake M. (2023). https://www.ft.com/content/c325fcdd-ab29-4cd3-9a74-6cc52b28ff5f?shareType=nongift.

[57] Mariani J., Eggers W.D., Kishnani P.K. (2023). https://www2.deloitte.com/xe/en/insights/industry/public-sector/ai-regulations-around-the-world.html.

[58] Levinson N. (2022). https://www.academia.edu/114982934/Inclusive_Anticipatory_Governance_Cyber_Technologies_Absorptive_Capacities_and_The_Case_of_the_United_Nations_Open_Ended_Working_Group_re_ICTS.

[59] Sastry G., Dafoe A., Cremer C.Z., Weller A., Meier P., Socher R., Altman S., Kunduri S. (2024). Computing Power and the Governance of Artificial Intelligence. arXiv.

[60] Crawford K. (2024). Generative AI’s environmental costs are soaring — and mostly secret. Nature.

[61] Campbell N.D., Adams S. (2024). https://www.project-syndicate.org/commentary/ustr-withdrawing-support-for-wto-provisions-protecting-open-internet-by-natalie-dunleavy-campbell-and-stan-adams-2024-02.

[62] Harris J. (2023). https://www.theguardian.com/lifeandstyle/2023/may/22/there-was-all-sorts-of-toxic-behaviour-timnit-gebru-on-her-sacking-by-google-ais-dangers-and-big-techs-biases.

[63] Harari Y.N. (2023). Yuval Noah Harari Argues That AI Has Hacked the Operating System of Human Civilisation. The Economist.

[64] Pauwels E. (2020). The Anatomy of Information Disorders in Africa.

[65] Gilbert M. (2005). Shared Values, Social Unity, and Liberty. Publ. Aff. Q..

[66] Vermeer M.J.D. (2024). https://www.rand.org/pubs/research_reports/RRA3408-1.html.

[67] Kerry C. (2024). https://www.brookings.edu/articles/the-good-the-not-so-good-and-the-ugly-of-the-uns-blueprint-for-ai/?b=1.

[68] Pouget H., Dennis C., Batemen J., Trager R.F., Araujo R., Belfield H., Cleeland B., Estier M., Futerman G., Guest O. (2024). https://carnegieendowment.org/research/2024/08/the-future-of-international-scientific-assessments-of-ais-risks?lang=en.

[69] Gwagwa A. (2024). https://www.chathamhouse.org/2024/06/artificial-intelligence-and-challenge-global-governance/06-resisting-colonialism-why-ai.

